# Sketches of the Medical Topography of Gibraltar

**Published:** 1830-07-01

**Authors:** 


SKETCHES OF THE MEDICAL TOPOGRAPHY OF THE MEDITERRANEAN: COMPRISING AN ACCOUNT OF GIBRALTAR, THE IONIAN ISLES, AND MALTA, &c. By *John Hennen*, M.D. &c. &c. Edited by his Son, *J. Hennen*, M. D. Octavo, pp. 666. Underwoods, 1830.

BY a paper published nine years ago in our esteemed contemporary, the Edinburgh Medical and Surgical Journal, it will be seen that the late Dr. Hennen directed his attention, in an especial manner, to MEDICAL TOPOGRAPHY. His subsequent residence, as inspector of hospitals, in Gibraltar, Malta, and the Ionian Islands, gave him ample field for indulging his favourite studies, and prosecuting his useful researches. The ample volume before us contains the result of his indefatigable labours, and, in conjunction with his "Principles of Military Surgery," will transmit his name to posterity, in the medical annals of his country.

### GIBRALTAR.

Passing over the introductory memoir, re-published from the Edinburgh Journal, we shall select for the present article the medical topography of Gibraltar, one of the strongest and most important fortifications in the possession of Britain—the key of the Mediterranean—and a severe thorn in the side of Spain.

This celebrated Pillar of Hercules juts out into the sea, in an oblong form, with a craggy ridge for its summit, more than fourteen hundred feet high—the rock being about three miles in length by half or three quarters of a mile in breadth. Its western side consists of a series of rugged slopes interspersed with abrupt precipices—its northern extremity, facing the neutral ground, is perfectly perpendicular, with the exception of a narrow ribbon along the shore. The eastern side is a range of precipices—while its southern point falls down into two small flats, called Windmill Hill and Europa Point. It is connected with Spain by a low sandy flat, called the Neutral Ground.

Passing over a number of minute topographical details, we shall now advert to some of the numerous sources of insalubrity which this singular fortress presents.

"The immediate neighbourhood of the spots now enumerated, demands the particular attention of the Medical Topographer. At each of them, public sewers discharge themselves, and public necessaries are erected; from these causes, as well as from the occasional admixture of marine exuviæ, the effluvia which arise are frequently very offensive, especially when the westerly winds blow, which drive them inwards upon the town, from which they are prevented from escaping with sufficient rapidity, by the intervention of the rock behind. In summer, when the afternoon sun lies for so many hours on the western face of the mountain, this nuisance is occasionally felt with peculiar severity.

"Besides these general nuisances, each of these spots has its own peculiarities. The offensive matters thrown up on the beach from the numerous small craft which are crowded around the vicinity of the 'Old Mole,' must tend to deteriorate the purity of the air in no small degree; much of these exuviæ are carried away daily, but much remain afloat, and when old hulks, timber, boats, and other incumbrances are allowed to lie on the beach, a considerable quantity of filth accumulates among them beyond the reach of the scavengers. When it is recollected, that the floating population of the Bay of Gibraltar may be estimated at 2000 souls the year round, the amount of animal and vegetable offal must obviously be considerable.

"To the north and south of the King's Bastion, several public sewers empty themselves, but not having been carried into the sea, or even to low water mark, a great proportion of their contents is left on the beach. It is only since the administration of Sir George Don that they have been carried as far as they are at present, but it is proposed to extend them so as to obviate the nuisance completely. Wooden sheds are also projected from the Line Wall in this neighbourhood, and serve as necessaries; the soil is never effectually removed from them, as the operation of the tide is not sufficiently powerful, even when it is at the highest. These sheds will soon, I trust, be removed, or so altered, that the soil may be at once conveyed into appropriate drains, or drop directly into the sea. From the Old Mole to the King's Bastion, nearly one-half of the Line Wall is covered by the breakwater already noticed. This breakwater was thrown up about the year 1788; within it, very extensive new works are now constructing. Until of late years the water had not free course, and the fœtor was excessive; even now it is at times very unpleasant, and is increased by some extensive sewers emptying themselves in the neighbourhood." 11.

The Camber near the Mole receives the contents of several sewers, and was notorious for bad smells a few years ago; but is less so now.

"In Rosia Bay, the sea is frequently, during the summer season, as stagnant as in a mill-pond. On the north end it is protected from the wind by high rocks; on the south it is defended by the Mole, and is only open to the westerly winds, which are most prevalent in winter. From the Line Wall, which runs along the rocks, two wooden necessaries, similar to those near the King's Bastion, project, and the soil is in like manner retained on the sandy beach. Two large sewers also empty themselves here. Exhalations of a very offensive nature arise from these sources, and with other circumstances, hereafter to be noticed, may tend to account for the unhealthy character which Rosia Bay has laboured under. In every spot where sewers mix their contents with the sea-water, numerous air-bubbles are perceptible on the surface, doubtless from the extrication of gas from the putrid matters thus accumulated. At the Old Mole this can be very readily observed. Mr. Hugh Fraser and Mr. P. Wilson, gentlemen of the Civil Hospital, from whose local knowledge I have derived much information, assure me that there is not a summer in which watermen, who sleep in their boats anchored at that part of the Mole where several sewers intermix their contents, are not seized with bilious remittents." 12.

About three miles from the garrison a small stream opens into the bay, and the banks are malarious, the inhabitants being subject to agues. At the distance of five miles there is a still more fertile source of fevers, at the mouth of the Guadaranque, where the ground is swampy and unhealthy. But again;—along the edge of the beach there is erected a causeway leading out to the Neutral Ground; and bounded by this causeway on the West, and by a part of the rock and Spain on the East and South, "there is an artificial inundation, which serves as a strong protection to the works."

"This inundation was formerly a morass, and the only one that has probably ever existed near the garrison. In plans of the fortress and of the seige of 1704, this morass is represented as communicating with the sea by a long narrow channel running parallel with the beach for some distance. In 1732 it was dug two feet below the level of low-water mark in the bay, and many deep pits in the quincunx form were sunk in it. At present it contains eight transverse and one longitudinal ditch, from ten to twelve feet deep; it covers nine acres of ground, and the depth of water generally, is from four to six feet. The contents can be partially let off by means of sluices, at the foot of the glacis; around the edges, however, the water is often dried up, leaving behind a green mossy substance, probably the remains of 'Lemnæ, Algæ,' and other aquatic plants, which, from their approach to an animal nature, and their abundance of nitrogen, are capable of affording highly putrescent miasmata. It also must partially stagnate in the pits and ditches above described; and its vicinity to the beach, behind the Mole, most probably serves to render the exhalations from this neighbourhood of a still more insalubrious tendency than they otherwise would be. To heighten all, there formerly existed a line of necessaries in the 'Orillon Ditch,' or Lazaretto, which, previous to 1814, discharged their soil into the inundation, and emitted a most offensive odour; they are now removed." 14.

Underground springs pour forth their contents, and dilute the sea-water of this inundation; while the rains that fall from the heavens percolate through the mountain and form supplies for wells and aqueducts. The whole surface of the mountain abounds in caves, fissures, and pot-like holes, which retain the rains, dews, and passing vapours. The most celebrated of these is St. Michael's Cave, more than eleven hundred feet above the sea, and whose bottom has never yet been ascertained.

"Wherever artificial excavations have been made in the rock, water, in almost every instance, filters through in great abundance from various springs and mountain rills, and in the Lines a very fine well is sunk, which was first discovered while the excavations were cutting." 16.

The public tanks, which are seven in number, are capable of holding 1,552,700 gallons of water! There are 105 private tanks.

"Although the rain pours in torrents down the entire western face of the mountain, there are several points at which these torrents have worn out deep channels or gullies for themselves. Five of them are particulaaly worthy of notice. Of this number four discharge their contents through the town, and one upon a large bank of sand to the southward of the New Alamada, close to the reservoir of the aqueduct. It should be kept in mind, that the points at which the rains pour off with the *greatest* precipitation, are precisely the points where there are no habitations; and that where the violence of the torrent ceases, there the residences of man commence, and there, consequently, aqueous exhalation is most felt by him." 19.

The bottoms and sides of all these gullies are rocky. In their general appearance, they resemble, on a small scale, the unhealthy "*fiumares*" of Sicily and the Ionian Isles, but without the vegetation which so richly clothes their banks. They run their course in the direction of the blue barracks, city, Mill-lane, Boyd's-buildings, and other spots notoriously unhealthy during the epidemic years 1804-10-13 and 1814. We cannot follow our observant author through his delineation of various localities on this celebrated rock, which are unquestionably capable of affording all the materials, in proper seasons, of febrific miasmata. But we must dedicate a page or two to the Neutral Ground, which, says Dr. H. is of *more* importance to the health of the garrison than is generally supposed.

This isthmus, which is a kind of quick-sand, has been occasionally washed over, to the extent of two-thirds of its surface, when strong easterly winds and spring tides prevailed in the winter time. Hence extensive pools have been left behind, close up to the gardens. The present governor has erected a dike to obviate these occurrences.

"In winter the rain-water forms numerous and extensive pools, which continue during the spring months. These pools are only dried up completely by the summer heats; but there is one near the high-road beyond the gardens which is never completely dry. Besides these adventitious depositions of moisture, there are numerous internal sources of permanent supply at the Neutral Ground. I have been led particularly to examine this spot, in consequence of the assertion that no source whatever of Marsh miasmata existed at Gibraltar. If, by this assertion, it is meant that *no morasses* at present exist, I perfectly concur with it, but I can go no further, because, under the present head, I have indicated numerous sources of aqueous exhalations, and I am about to point out others of a still more extensive nature. Indeed, the most superficial observer could scarcely ride over the Neutral Ground without perceiving many external evidences of underground moisture. The 'Arundo Phragmites,' the infallible test, grows even now, in great luxuriance, on several of the banks which surround the gardens; it is only necessary to thrust the reeds into the sand and they soon take root. It appears that this plant was formerly much more common and more extensive, for in the different histories of the seige, we frequently meet with accounts of the reeds being set on fire, &c.; but the fact does not rest on this species of evidence, or on external appearances, the auger and the shovel prove it completely." 22.

At almost every spot that has hitherto been perforated, water has been found within six feet of the surface, often much nearer. The quantity of water drawn from the Neutral Ground is immense. The gardens alone consume one thousand gallons daily, independent of what is used for domestic purposes and the shipping.

From thermometrical observations made during ten years, viz. from 1816 to 1826, the medium range of Fahrenheit in the months of June, July, August, and September, was 84 degrees—the medium range of the East and West Indies. In the months of January, February, and December, it was 51 degrees. The Easterly winds, called Levanters, are peculiarly disagreeable and unwholesome.

"While the easterly winds blow the sewers throughout the town emit the most offensive vapours, and even before they come on, the practised olfactory organs of the inhabitants detect their approach. Is this the result of the humid atmosphere softening the soil of the sewers, and occasioning an increased exhalation?" 32.

We regret that our limits will not permit us to do full justice to the minute and highly interesting medico-topographical details contained in this section of Dr. Hennen's work. Suffice it to say that the sources of unhealthy exhalations are exceedingly numerous on this rock—a circumstance which several months residence at Gibraltar many years ago, strongly impressed on our minds. Intermittent and remittent fevers, dysenteric affections, and infantile marasmus are the worst diseases among the inhabitants. And after all the attention which has been paid to the increase of salubrity in Gibraltar, there are still abundant sources of sickness left—some of which are beyond the reach of human remedy.

"Stables and sheds for the labouring cattle, and for some of the poorer inhabitants are scattered along the side of the hill; from these and from several of the better habitations, offal is deposited in or upon the banks of the gully. This offal consists of the most offensive materials, the situation not being very accessible, and but little frequented by the inspectors of nuisances. These matters often lie where they are deposited, until the lighter parts are dispersed by the winds, and the more solid are hardened into an uniform mass. When the rains fall, the more soluble parts of this compost are carried down in a state of minute division, while the grosser are forced along 'en masse,' and are thrown into corners and hollows along the course of the stream, or hurried into the sewers, where they mingle with, and often obstruct the course of the waters." 49.

It is an erroneous supposition that the surface of Gibraltar is so precipitous that all the water which falls from the skies is immediately carried off. "That part of the town which runs from the main street to the Line-Walk, is naturally as level as most towns in England." Many parts are what may be called "made ground," which absorb the rain, and retain a considerable portion of it, "the effects of which, when it is saturated with putrid materials, such as are enumerated above, cannot but be influential on the salubrity of the neighbourhood." The filth, and perhaps the insalubrity of Gibraltar is increased by the system of washing clothes in the houses of the inhabitants, the foul waters thus produced being thrown into sewers or the adjoining gutters, there to annoy the olfactories and augment the putrefactive process.

The neighbourhood of the dock-yards, the naval gardens, and Rosia Bay, have always been considered as situations capable of emitting febrific miasmata, such as the exhalations which arise from extensive gardens highly cultivated—from a beach that lies low, receiving common sewers—and from a neighbourhood on which such a quantity of rain falls as to supply an extensive set of tanks, capable of holding more than a million gallons of water. The following passage deserves attention.

"It has been stated, that the vapours of sewers and cesspools were evidently not so pernicious as was apprehended, because the fever did not follow the line of sewer, *topographically*, and because several sewers were afterwards opened without generating fever in the immediate neighbourhood. But it is not the neighbourhood of a *good* sewer, or of one *fully opened* up, that is injurious to health; the injury is done by an *inefficient* sewer, the vapours of which are not diluted and neutralized by the free access of atmospheric air. A bad sewer, in a confined place, is worse than no sewer at all, because it serves to collect, accumulate, and retain filth; while a line of open drain, in a situation fully exposed to the air, will be comparatively innocuous as a source of febrific miasmata. Free ventilation makes all the difference; a partial rent in a sewer is always found to be more injurious than an opening of several yards, made for the purpose of repair. It should also be remembered, that a close sewer emits no vapours along its *topographical course*: they proceed from its mouth only, or from accidental rents." 52.

Population has made rapid advances during the last 30 or 40 years in Gibraltar. In 1791 it was 2885—in 1826 it amounted to 15,480!! When the military population is added, the whole exceeds 20,000 souls. The poorer classes are crowded together in apartments of a very bad description—the majority of them being "strikingly deficient in size, ventilation, and the means of cleanliness," while some of them "are utterly unfit for human habitations."

"In the premises of a Jew, in 'Victualling-office-lane,' I found, on a ground floor, seven occupied apartments—one store-room, and one necessary, built around an area of twenty-five feet by seventeen feet five inches; this area was encumbered with casks, baskets, and jars piled along the walls, and the upper part was curtailed by a projecting gallery, so that the space left for ventilation was reduced to eight feet five inches by five feet six." 70.

When it is considered that the summer heat of Gibraltar is 80 degress of Fahrenheit, nearly that of the Tropics, some idea may be formed of the insalubrity of such dwellings as those described by Dr. Hennen. The foregoing abode contained 24 individuals, and of the occupied apartments, only two had windows to the street—two had small slits in the upper part of the wall—the others had neither air nor light, except what they derived from the area. Upon the whole, though Gibraltar is improved beyond all expectation or conception by the present Governor, it is still a place "confined and ill-ventilated, in which innumerable obstacles to cleanliness exist, and with a population filthy in themselves, and over-crowded perhaps beyond any other community in the world."

Dr. Hennen not having been present in any of the severe epidemics of 1804—10—13, and 14, declines giving an opinion on the important and much litigated points of endemic or imported fevers.

"The professional characters of the supporters of the opposite theories stand high, and I neither question the fidelity of their reports, (to the best of their knowledge,) nor the uprightness of their intentions; but it has been long allowed by the more dispassionate part of the profession, that much is assumed on defective evidence; that much special pleading has been entered into, and that there is often room to suppose, that the opposite parties have contended more for victory than truth." 92.

The indefatigable author then gives an impartial and strictly neutral sketch of the different epidemics, from 1804 onwards. By this it appears that the immunity from second attacks was ascertained 26 years ago.

"Relapses were frequent about the end of October and beginning of November, principally among intemperate subjects; but either no instance of second attack occurred, or if it did, it formed an exception to a rule, admitted on all hands to be general. The same was observed of persons who had had the yellow fever in the West Indies, and advantage was taken of the circumstance in conducting the duties, both in the hospitals and in the barracks. A quarantine encampment, of those who had not passed through the fever, was formed on the 9th of November. These men, with the exception of the 13th regiment, took their bedding with them; the 13th, by the precaution of their Colonel, left their old dirty bedding behind, and brought two clean blankets in lieu: not a man of this corps was attacked with disease, whilst, on the 12th of this month, five men of other corps were seized, and within the three following days every regiment, except the 13th, had men taken ill. The bedding was then taken from them all, and fresh and clean articles supplied. I cannot discover whether any others were attacked after this change. In whatever light it may be taken by either the contagionists or non-contagionists, this fact suggests a useful precaution, which indeed it is astonishing could have been overlooked by the most careless observer." 110.

Our author concludes by expressing his creed in the words of Dr. Hancock—from which it will appear that Dr. H. is a contingent contagionist.

"'The conclusions,' says Dr. Hancock, 'to which I have referred, are these; that our continued fever, (whether called typhus, or synochus, appears to me quite immaterial,) often arises from small beginnings ; that it has a power, under certain circumstances, of generating a contagious '*seminium de novo*,' which is sometimes more, sometimes less easily disseminated; that the symptoms of the disease are liable to be aggravated to a considerable height by local causes, chiefly in the autumnal season, and still more remarkably, if it has prevailed as an epidemic in pestilential seasons ; and that it declines in winter to give place to its milder form, or to some other disease in the ensuing summer.'

"To these conclusions, I would add one more quotation from the same indefatigable author—'He that, exclusively believing in a contagious virus, asserts medicine and police regulations can do all, and attributes the removal of pestilence *solely* to their means, may be as much in error as he who, convinced of a general contamination in the air, denies contagion, and believes a crowded or scattered population would make no difference in the mortality, or that a filthy habitation would add nothing to the malignity of the distemper; and that, as the disease is from the air, it matters not whether he stands idly gazing on it, till it shall cease, or assists to remove a local nuisance out of the way.'" 117.

We regret to learn that Dr. Hancock himself fell a victim to the Gibraltar epidemic of 1828, while ardently engaged in attending the sick of the garrison, and investigating the nature of the fever.

We have no room left for noticing the common annual diseases of Gibraltar. But Dr. Hennen states that remittent fevers "are of frequent occurrence"—"and that genuine yellow fevers (such as are seen in the West Indies) accompanied with the true black-vomit, occur both in the Civil Hospital and in private habitations *every season*." Pulmonary affections are so frequent in Gibraltar that they have been styled the "TRUE ENDEMIC of the rock."

In our next article we shall introduce some interesting particulars respecting the medical topography of the Ionian Isles.

